# *Ent*-kaurane diterpenoids from the Annonaceae family: a review of research progress and call for further research

**DOI:** 10.3389/fphar.2023.1227574

**Published:** 2023-06-29

**Authors:** Traore S. Ibrahim, Purevdulam Khongorzul, Moses Muyaba, Raphael N. Alolga

**Affiliations:** ^1^ Department of Pharmacognosy, State Key Laboratory of Natural Medicines, School of Traditional Chinese Pharmacy, China Pharmaceutical University, Nanjing, China; ^2^ Department of Pharmaceutical Chemistry and Pharmacognosy, School of Pharmacy, Eden University, Lusaka, Zambia

**Keywords:** Annonaceae, ent-kaurane diterpenoid, biosynthesis, biological activity, isolation

## Abstract

The Annonaceae is one of the plant families with members that are credited with numerous pharmacological functions. Among the group of compounds responsible for these bioactivities are the *ent*-kaurane diterpenoids. The *ent*-kauranes are a group of 20-Carbon, tetracyclic diterpenoids that are widely distributed in other plant families including the Annonaceae family. This mini-review focuses mainly on the *ent*-kaurane diterpenoids isolated from the Annonaceae family, delineates the various biological activities of these compounds, and highlights the research gaps that exist for further scientific scrutiny.

## Introduction

A catalog of the Annonaceae family reveals that it comprises shrubs, climbers, aromatic trees and lianas that are almost ubiquitously distributed (i.e., found in almost all seven continents) (Al Kazman et al., 2022). This family is sometimes referred to as a “living fossil” due to the characteristic archaic and primitive features of member plants that have enabled their survival over the years (Attiq et al., 2017; Couvreur et al., 2022). This family comprises at least 120 genera and 2400 species widely distributed in four main subfamilies: Annonoideae, Anaxagoreoideae, Malmeoideae, and Ambavioideae (Attiq et al., 2017).

The classification of the Annonaceae family has undergone systematic evolution after the work of Dunal in 1817 (Attiq et al., 2017; Al Kazman et al., 2022). The work of Dunal was mainly based on the fruit morphology of member plants. Another system of classification was later established by Baillon (1868) and Diels and Alder (1932) on the basis of the floral characteristics of member plants (Attiq et al., 2017; Al Kazman et al., 2022). A more holistic approach that takes into consideration the floral characteristics and fruit morphology was propounded by Fries (1959) and currently serves as the gold standard for the classification of plants in this family (Attiq et al., 2017; Couvreur et al., 2022). Economically, members of this family have often served as a source of food and medicine for traditional uses. Notable members of this family include, *Xylopia aethiopica*, *Xylopia parvifolia*, *Annona muricata*, *Annona reticulata*, *Uvaria grandi*, *Cananga odorata*, *Friesodielsia latifolia*, *Anaxagorea dolichocarpus*, etc (Couvreur et al., 2022). Traditionally, these plants have been used for diverse therapeutic purposes such as, pain management and treatment of inflammation-related diseases (Almeida et al., 2012; Woode et al., 2012; Cercato et al., 2015). Phytochemical investigations have found a diversity in the bioactive compounds isolated from this family. The compounds range from alkaloids, flavonoids to acetogenins and *ent*-kauranes (Chan et al., 1993; Wu, 2006; Liaw et al., 2016; Zhao et al., 2022).

The *ent*-kauranes which are a group of structurally diverse tetracyclic compounds form an integral part of the bioactive compounds isolated from the Annonaceae family (Wu, 2006; Zhao et al., 2022). Structural diversity within the *ent*-kauranes is usually the result of changes to the parent skeleton such as bond cleavages, oxidation, intramolecular cyclization or structural rearrangements (Yang et al., 2002; Zhao et al., 2022). They are credited with biological functions including but not limited to antifungal, antibacterial, antitumor and anti-inflammatory activities (Wang et al., 2011; Zhao et al., 2022). This review seeks to throw light on the *ent*-kauranes diterpenoids with the view to directing the attention of researchers on the need for further research on this group of compounds. The content of this review will include thematic areas such as the biosynthesis, chemistry, and bioactivities of the *ent*-kaurane diterpenoids and call for further research.

## Methodology

Relevant published literature was retrieved from various databases such as Web of Science, Pubmed, google scholar, Elsevier, ACS using the following key words, singly or as combinations: *ent*-kaurane diterpenoids; Annonaceae; biosynthesis; biological activities. Publications on *ent*-kaurane diterpenoids from other plant families aside from Annonaceae were excluded. Only relevant publications in the English language were used. Publications in other languages such as Chinese (Mandarin) were also excluded. On the basis of this criterion and relevance to the topic, the articles were scaled down from a total of about 6,342 articles to 98. A flow chart of the methodology used is summarized in Supplementary Figure S1.

### Biosynthesis of the *ent*-kaurane diterpenoids

**FIGURE 1:**
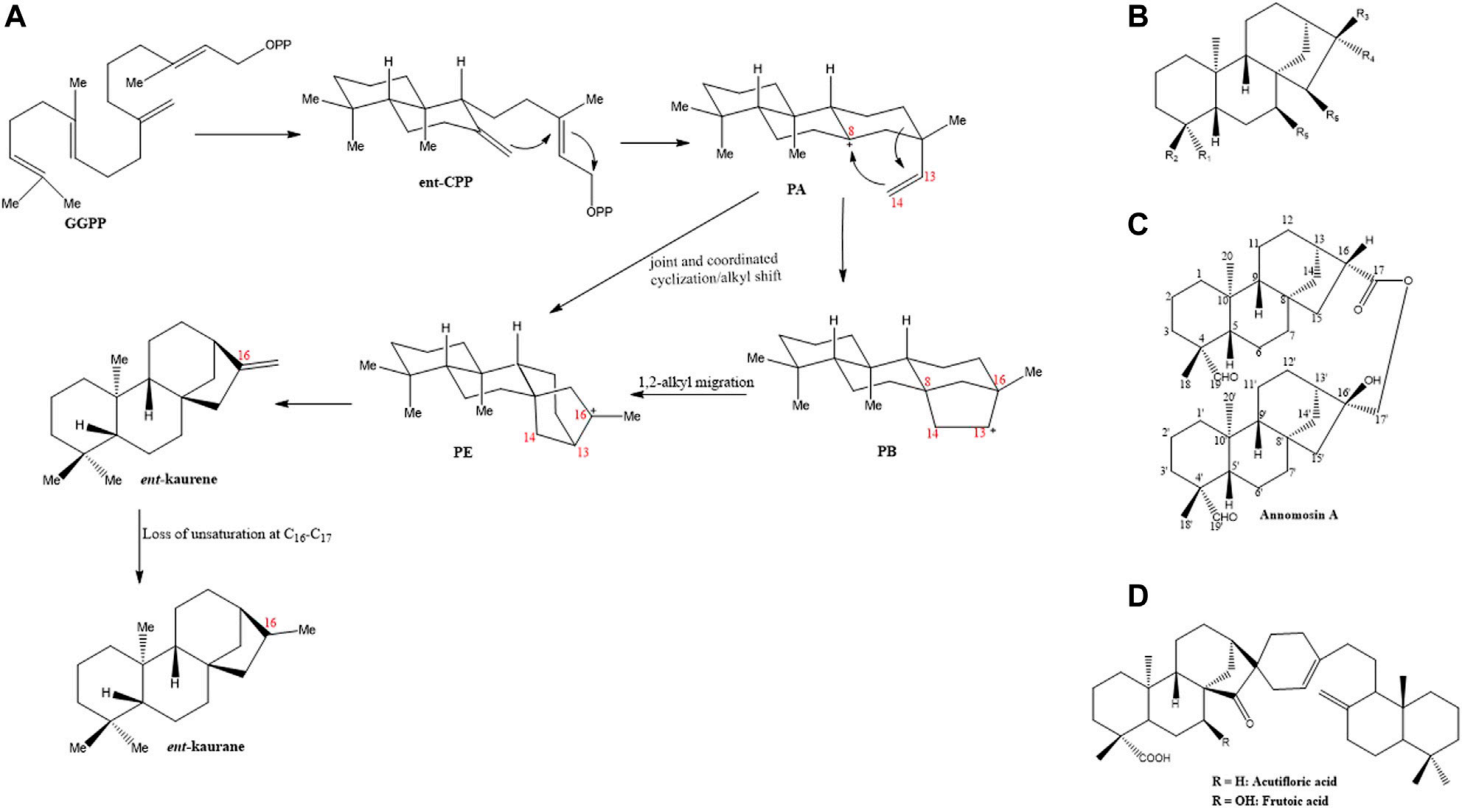
**(A)** Schematic outline of the biosynthetic pathway of *ent*-kaurene and *ent*-kaurane (adopted from Zhao et al., 2022). **(B)** Basic skeleton of the *ent*-kaurane diterpenoids **(C)** Chemical structure of dimeric *ent*-kaurane diterpenoid, Annomosin A **(D)**. Basic skeleton of acutifloric acid and frutoic acid, two dimeric *ent*-kaurane diterpenoids.

The term “*ent*” which stands for “*enantiomeric*” traces its roots to the earliest identified diterpene from the leaf oil of *Agathis*, a plant locally known in New Zealand as *Kauri pine* (Zhao et al., 2022). Due to its negative optical rotation, it was subsequently named “*ent*-kaurene”. The *ent*-kauranes, a group of 20-Carbon, tetracyclic diterpenoids are widely distributed in other plant families including the Asteraceae, Lamiaceae, Compositae, Euphorbiaceae, Pteridaceae families aside from the Annonaceae family (Aplin et al., 1963). They are generally accepted as intermediates in the biogenesis of growth hormones in the gibberellin plant (Yamaguchi, 2008; Hedden, 2020). Various strategies have been devised to synthetically produce some *ent*-kaurane diterpenoids as summarized by Zhao et al. (2022). However, in the parent plants, the *ent*-kauranes diterpenoids are biosynthesized from geranylgeranyl pyrophosphate (GGPP), a universally accepted precursor for diterpenes. Under the enzymatic action of copalyl diphosphate synthase, CPS (also called kaurene synthase A), GGPP is converted either to *ent*-copalyl diphosphate (*ent*-CPP) or syn-CPP based on the specificity of the enzyme (García et al., 2007). The action of *ent*-CPP synthase produces *ent*-CPP as the intermediate which is then converted in a series of steps to *ent*-kaurene by *ent*-kaurene synthase or kaurene synthase B. Mechanistically, *ent*-CPP undergoes a cascade of cyclization to produce a tricyclic intermediate PA that possesses a tertiary carbocation at C-8. The saturation at C-14 intramolecularly seeks this carbocation, the result of which generates a tetracyclic beyeranyl-13-cation intermediate, PB (i.e., carbocation is located at C-13). A more stable form of this intermediate (tertiary carbocation) is produced after a 1,2-alkyl migration to generate the ent-kaurenyl-16-cation PE. Alternatively, the tertiary carbocation PE can be directly formed from PA through the joint and coordinated cyclization and alkyl shift processes in a bid to avoid the formation of the less stable secondary carbocations (García et al., 2007; Zhao et al., 2022). *Ent*-kaurene is finally generated after proton removal from the tertiary carbocation thereby producing the required exocyclic alkene. Various chemical modifications of the parent *ent*-kaurene carboskeleton such as, C-C bond cleavage, oxidation or structural rearrangements result in the productions of different diterpenoids. For instance, the *ent*-kaurane carboskeleton is generated when the unsaturation at C-16 and C-17 is lost. These processes have been schematically summarized in Figure 1A.

### *Ent*-kaurane diterpenoids isolated from the Annonaceae family

On the basis of their structural characteristics, the *ent*-kaurane diterpenoids in general can be categorized into the seco-*ent*-kauranoids, the C-20 oxygenated *ent*-kauranoids, the C-20 non-oxygenated *ent*-kauranoids, the *nor*- or rearranged-*ent*-kauranoids and grayanes (Sun et al., 2006; Liu et al., 2017). For research on the isolation and structural elucidations of *ent*-kaurane diterpenoids from plants within the Annonaceae family, the group of Prof. Yang-Chang Wu have contributed enormously. Their series of works on the Formosan Annonaceous plants deserve commendation (Wu, 2006). On the whole, at least 70 *ent*-kaurane diterpenoids have been isolated and structurally characterized from plants belonging to the Annonaceae family (Eshiet et al., 1971; Etse et al., 1987; Wu, 2006; Nhiem et al., 2015). Table 1 summarizes some of the *ent*-kaurane diterpenoids and the plants from which they were isolated, Figure 1B illustrates their basic skeletal structure while the chemical structures of all compounds tabulated (Table 1) are shown in Supplementary Figure S2. These compounds range from simple *ent*-kaurane/*ent*-kaurene diterpenes and derivatives of same to dimeric diterpenoids. Most of the compounds were isolated from various *Annona* (Wu et al., 1996; Chang et al., 1998; Chen et al., 1998; Chen et al., 2000; Yang et al., 2002) and *Xylopia* species (Hasan et al., 1982; 1985; Lajide et al., 1995; Takahashi et al., 1995; Désiré et al., 2013). Of all the compounds highlighted, annomosin A (16β-hydroxy-19-al-*ent*-kauran-17-yl-16β-hydro-19-al-*ent*-kauran-17-oate) is dimeric in nature, the first of its kind reported in the Annonaceae family (Wu, 2006). It is composed of two *ent*-kaurane monomeric units, thus, 19-al-*ent*-kauran-17-oic acid and 16,17-dihydroxy-*ent*-kauran-19-al. Other dimeric *ent*-kaurane diterpenoids which were isolated from the *Xylopia acutiflora* specie, thus, acutifloric acid and frutoic acid, are composed of a labdane monomer and an *ent*-kaurane monomer (Hasan et al., 1985; Takahashi et al., 1995) (Figures 1C, D).

**TABLE 1:** List of non-dimeric *ent*-kaurane diterpenoids isolated from plants in the Annonaceae family.

No.	Compound name	Substituents						Plant source	References
		R1	R2	R3	R4	R5	R6		
1	16α-hydro-*ent*-kauran-17,19-dioic acid	CH_3_	COOH	COOH	H			*A. glabra, R. mucosa*	Wu (2006)
2	*ent*-kaur-16-en-19-oic acid (known as Kaurenoic acid)	CH_3_	COOH	Δ16, 17				*A. squamosa, A. glabra, A. cherimola, X. aethiopica*	Wu, 2006; Eshiet & Akisanya 1971
3	16β-hydro-*ent*-kauran-17, 19-dioic acid	CH_3_	COOH	H	COOH			*A. squamosa*	Wu (2006)
4	16β-hydroxy-17-acetoxy-*ent*-kauran-19-oic acid	CH_3_	COOH	OH	CH_2_OAc			*A. squamosa, A. glabra, A. cherimola*	Wu (2006)
5	16α-hydroxy-*ent*-kauran-19-oic acid	CH_3_	COOH	CH_3_	OH			*A. glabra, Xylopia acutiflora*	Wu, 2006; Hassan et al., 1982
6	16α-hydro-19-al-*ent*-kauran-17-oic acid	CH_3_	CHO	COOH	H			*A. squamosa, A. glabra*	Wu (2006)
7	16β-hydro-17-hydroxy-*ent*-kauran-19-oic acid	CH_3_	COOH	H	CH_2_OH			*A. squamosa, A. cherimola, A. reticulata*	Wu, 2006; Chen et al., 1998
8	16β-hydro-17-acetoxy-*ent*-kauran-19-oic acid	CH_3_	COOH	H	CH_2_OAc			*A. squamosa, A. cherimola*	Wu (2006)
9	16β,17-dihydroxy-*ent*-kauran-19-oic acid	CH_3_	COOH	OH	CH_2_OH			*A. squamosa, A. glabra, X. frutescens*	Wu, 2006; Takahashi et al., 1995
10	16α-hydro-17-hydroxy-*ent*-kauran-19-oic acid	CH_3_	COOH	CH_2_OH	H			*A. squamosa, A. cherimola, A. reticulata*	Wu, 2006; Chen et al., 1998
11	16α-hydro-17-acetoxy-*ent*-kauran-19-oic acid	CH_3_	COOH	CH_2_OAc	H			*A. reticulata, A. cherimola*	Wu, 2006; Chen et al., 1998
12	16α,17-dihydroxy-*ent*-kauran-19-oic acid	CH_3_	COOH	CH_2_OH	OH			*A. squamosa, A. glabra, A. reticulata*	Wu (2006)
13	16α-methoxy-*ent*-kauran-19-oic acid	CH_3_	COOH	CH_3_	OCH_3_			*A. glabra*	Wu (2006)
14	16β,17-diacetoxy-*ent*-kauran-19-oic acid	CH3	COOH	OAc	CH_2_OAc			*A. glabra*	Wu (2006)
15	17-hydroxy-*ent*-kaur-15-en-19-oic acid	CH_3_	COOH	Δ15, 16	CH_2_OH			*A. glabra*	Wu (2006)
16	methyl-16α-acetoxy-17-oate-*ent*-kauran-19-oic acid	CH_3_	COOH	COOCH_3_	OAc			*A. glabra*	Wu (2006)
17	*ent*-kaur-15-en-17,19-diol	CH_3_	CH_2_OH	Δ15, 16	CH_2_OH			*A. glabra*	Wu (2006)
18	16α-hydro-19-ol-*ent*-kauran-17-oic acid	CH_3_	CH_2_OH	COOH	H			*A. glabra*	Wu (2006)
19	methyl-16α-acetoxy-19-al-*ent*-kauran-17-oate	CH_3_	CHO	COOCH_3_	OAc			*A. glabra*	Wu, 2006; Chang et al., 1998
20	16β-hydroxy-17-acetoxy-*ent*-kauran-19-al	CH_3_	CHO	OH	CH_2_OAc			*A. squamosa, A. glabra, A. cherimola*	Wu (2006)
21	16β, 17-dihydroxy-*ent*-kauran-19-al	CH_3_	CHO	OH	CH_2_OH			*A. squamosa*	Wu (2006)
22	16α-hydro-17-hydroxy-*ent*-kauran-19-al	CH_3_	CHO	CH_2_OH	H			*A. cherimola*	Wu, 2006; Chen et al., 1998
23	16α-hydro-19-acetoxy-*ent*-kauran-17-oic acid	CH_3_	CH_2_OAc	COOH	H			*A. glabra, R. mucosa*	Wu, 2006; Chang et al., 1998
24	*ent*-kauran-16, 17, 19-triol	CH_3_	CH_2_OH	OH	CH_2_OH			*A. squamosa, A. reticulata*	Wu (2006)
25	*ent*-kauran-16α-ol	CH_3_	CH_3_	CH_3_	OH			*X. acutiflora, X. aethiopica, A. senegalensis*	Hassan et al., 1982; Lajide et al., 1995
26	*ent*-kaur-16-en-19-ol	CH_3_	CH_2_OH	Δ16, 17				*A. squamosa, A. glabra, X. frutescens*	Wu, 2006; Takahashi et al., 1995
27	methyl-16β-acetoxy-19-al-*ent*-kauran-17-oate	CH_3_	CHO	OAc	COOCH_3_			*A. glabra*	Wu (2006)
28	16α,17-dihydroxy-*ent*-kauran-19-al	CH_3_	CHO	CH_2_OH	OH			*A. squamosa*	Wu (2006)
29	methyl-16α-hydro-19-al-*ent*-kauran-17-oate	CH_3_	CHO	COOCH_3_	H			*A. glabra*	Wu (2006)
30	16β-hydro-17-hydroxy-*ent*-kauran-19-al	CH_3_	CHO	H	CH_2_OH			*A. squamosa, A. cherimola*	Wu, 2006; Chen et al., 1998
31	16β-hydroxy-17-acetoxy-18-nor-*ent*-kauran-4β-hydroperoxide	OOH	CH_3_	OH	CH_2_OAc			*A. squamosa*	Wu, 2006; Yang et al., 2002
32	16β-hydroxy-17-acetoxy-19-nor-*ent*-kauran-4α-formate	CH_3_	OCHO	OH	CH_2_OAc			*A. squamosa*	Wu, 2006; Yang et al., 2002
33	16β,17-dihydroxy-18-nor-*ent*-kauran-4β-hydroperoxide	OOH	CH_3_	OH	CH_2_OH			*A. squamosa*	Wu, 2006; Yang et al., 2002
34	16α-hydro-17-hydroxy-19-nor-*ent*-kauran-4α-ol	CH_3_	OH	CH_2_OH	H			*A. squamosa*	Wu, 2006; Yang et al., 2002
35	19-nor-*ent*-kauran-4α, 16β, 17-triol	CH_3_	OH	OH	CH_2_OH			*A. squamosa*	Wu (2006)
36	16α-hydro-*ent*-kauran-17-oic acid	CH_3_	CH_3_	COOH	H			*A. glabra*	Wu (2006)
37	methyl-16β, 17-dihydroxy-*ent*-kauran-19-oate	CH_3_	COOCH_3_	OH	CH_2_OH			*A. reticulata*	Wu, 2006; Etse et al., 1987
38	16β-hydroxy-17, 19-diacetoxy-*ent*-kaurane	CH_3_	CH_2_OAc	OH	CH_2_OAc			*A. cherimola*	Wu (2006)
39	16β-acetoxy-17-hydroxy-19-nor-*ent*-kauran-4α-ol	CH_3_	OH	OAc	CH_2_OH			*A. squamosa*	Wu, 2006; Yang et al., 2002
40	methyl-16α-acetoxy-19-nor-*ent*-kauran-4α-ol-17-oate	CH_3_	OH	COOCH_3_	OAc			*A. glabra*	Wu et al., 2006
41	19-nor-*ent*-kauran-4α-ol-16α-hydro-17-oic acid	CH_3_	OH	COOH	H			*A. squamosa, A. glabra*	Wu (2006)
42	16β-hydro-*ent*-kauran-17-oic acid	CH_3_	CH_3_	H	COOH			*A. glabra*	Wu (2006)
43	dimethyl-16α-hydro-*ent*-kauran-17, 19-dioate	CH_3_	COOCH_3_	COOCH_3_	H			*A. glabra*	Wu (2006)
44	7β,16α,17-trihydroxy-*ent*-kauran-19-oic acid	COOH	CH_3_	CH_2_OH	OH	OH		*A. glabra*	Nhiem et al., 2014
45	7β,17-dihydroxy-16α-*ent*-kauran-19-oic acid 19-O-β-D-glucopyranoside ester	COOGlc	CH_3_	CH_2_OH		OH		*A. glabra*	Nhiem et al., 2014
46	15-oxo-*ent*-kaur-16-en-19-oic acid	COOH	CH_3_	CH_2_			O	*X. aethiopica*	Soh et al., 2022; Lajide et al., 1995
47	*ent*-7-oxo-kaur-16-en-19-oic acid	COOH	CH_3_	CH_2_		O		*X. aethiopica, X. sericea*	Lajide et al., 1995; Gontijo et al., 2019
48	7β-acetoxy-*ent*-kaur-16-en-19-oic acid	COOH	CH_3_	CH_2_		OAc		*X. acutiflora, X. aethiopica*	Hassan et al., 1982; Lajide et al., 1995
49	7β-hydroxy-*ent*-kaur-16-en-19-oic acid	COOH	CH_3_	CH_2_		OH		*X. aethiopica*	Hassan et al., 1982
50	15β-acetoxy-*ent*-kaur-16-en-19-oic acid (known as Xylopic acid)	COOH	CH_3_	CH_2_			OAc	*X. aethiopica*	Lajide et al., 1995
51	*ent*-kauran-16α-19-diol	CH_2_OH	H	CH_3_	OH			*X. aethiopica*	Lajide et al., 1995
52	*ent*-16β-hydroxy-kauran-19-oic acid	COOH	CH_3_	OH	CH_3_			*X. frutescens*	Takahashi et al., 1995
53	*ent*-16β-hydroxy-kaurane	CH_3_	CH_3_	CH_3_	OH			*X. frutescens*	Takahashi et al., 1995
54	16,17-epoxy-15-oxo-*ent*-kauran-19-oic acid	COOH	CH_3_	CH_2_O			O	*X. aethiopica*	Soh et al., 2022
55	16α-acetoxy-*ent*-kauran-19-al-17-methyl ester	CHO	CH_3_	COOCH_3_	OAc			*A. glabra*	Chen et al., 2000
56	16α-acetoxy-19-nor-*ent*-kauran-4α-ol-17-methyl ester	OH	CH_3_	COOCH_3_	OAc			*A. glabra*	Chen et al., 2000
57	16α-hydro-*ent*-kauran-17,19-dimethyl ester	COOCH_3_	CH_3_	COOCH_3_	H			*A. glabra*	Chen et al., 2000
58	16α-acetoxy-*ent*-kauran-19-oic acid-17-methyl ester	COOH	CH_3_	COOCH_3_	OAc			*A. glabra*	Chen et al., 2000
59	l6β-hydroxy- 17, 19-diacetoxy-*ent*-kaurane	CH_2_OAc	CH_3_	OH	CH_2_OAc			*A. cherimola*	Chen et al., 1998
60	*ent*-kauran-19-al-17-oic acid	CHO	CH_3_	COOH	H			*A. senegalensis*	Eshiet & Akisanya 1971
61	19-nor-kauran-4α-ol-17-oic acid	OH	CH_3_	COOH	H			*A. senegalensis*	Eshiet & Akisanya 1971
62	*ent*-16α, 17-dihydroxy-kaurane	CH_3_	CH_3_	CH_2_OH	OH			*A. reticulata*	Etse et al., 1987
63	*ent*-16β, 17-dihydroxy-kaurane	CH_3_	CH_3_	OH	CH_2_OH			*A. reticulata*	Etse et al., 1987
64	methyl-16β-hydroxy-17-acetoxy-*ent*-kauran-19-oate	COOCH_3_	CH_3_	OH	CH_2_OAc			*A. reticulata*	Etse et al., 1987
65	methyl-16β, 17-diacetoxy-*ent*-kauran-19-oate	COOCH_3_	CH_3_	OAc	CH_2_OAc			*A. reticulata*	Etse et al., 1987
66	16α-hydroxy-17-acetoxy-*ent*-kauran-19-al	CHO	CH_3_	OH	CH_2_OAc			*A. squamosa*	Wu et al., 1996
67	19-nor-*ent*-kaurane-4α,16β-17-triol	OH	CH_3_	OH	CH_2_OH			*A. squamosa*	Wu et al., 1996

A, *annona*; X, *xylopia*.

### Biological activities

In spite of the fact that a lot of the *ent*-kaurane diterpenoids have been isolated and reported in literature, not much biological evaluations have been done on them. The few compounds that have been assessed are reported to possess a vast array of biological activities including anti-inflammatory (Yeh et al., 2005; Nhiem et al., 2015), antimicrobial (Boakye-Yiadom et al., 1977), anti-HIV (Wu et al., 1996; Chang et al., 1998), anticancer (Fatope et al., 1996), termite antifeedant (Lajide et al., 1995), hypotensive and coronary vasodilatory (Somova et al., 2001) and anti-platelet aggregation (Yang et al., 2002) effects. Only two of these compounds, thus, kaurenoic acid, KA (Supplementary Figure S3A) and xylopic acid, XA (Supplementary Figure S3B) have received a considerable amount of biological scrutiny by many a researcher. A summary of the biological activities of these two compounds is therefore highlighted herein.

### Kaurenoic acid (KA)

KA (*ent*-kaur-16-en-19-oic acid) has been credited with a plethora of biological activities. It has been reported to attenuate inflammatory processes via diverse mechanisms. Its anti-inflammatory effects have been partly attributed to its ability to activate the transcription factor, nuclear factor erythroid 2-related factor 2, Nrf2 (Paiva et al., 2002; Lyu et al., 2011; Kim et al., 2016), downregulate Th2 and NF-κB/cytokine-related pathways (Borghi et al., 2022) and the transforming growth factor-β (TGF-β) signaling (Kim et al., 2017). It was also reported to dose-dependently inhibit prostaglandin E2 release, nitric oxide (NO) production, inducible nitric oxide synthase (*i*NOS) and cyclooxygenase-2 (COX-2) expressions (Choi et al., 2011). KA has also been reported to exhibit antinociception in various pain models (Dalenogare et al., 2019; Montiel-Ruiz et al., 2020; Zaninelli et al., 2023). Its analgesic effect has been linked to underlying mechanisms such as the inhibition of cytokine production and NO-cyclic GMP-protein kinase G-ATP-sensitive potassium channel signaling pathway activation (Mizokami et al., 2012).

The potential of KA against diverse microbes has been reported. Together with five of its derivatives, de Andrade et al. (2011) assessed its anticariogenic activity and reported its bactericidal effect against *Streptococcus mutans*, the primary causative agent of dental caries (Moreira et al., 2016; Moon et al., 2022). Martins et al., who investigated 12-kaurane-type diterpenes for their antibacterial effects against a group of bacteria that cause endodontic infections reported satisfactory activities for KA and its salt (Martins et al., 2018). On the basis of their proteomic data, they inferred that the possible mechanisms that underlie these antibacterial effects could be due to the ability of KA and its salt to hamper bacterial metabolism and virulence factor expression (Martins et al., 2018). KA has been found to be effective against other Gram-positive bacteria such as *Bacillus cereus* (Wilkens et al., 2002), and *Staphylococcus aureus* (Okoye et al., 2012; Pereira et al., 2012; Arciniegas et al., 2018). Additionally, KA was found to demonstrate good antifungal activity against *Epidermophyton floccosum*, *Trichophyton rubrum* and *Trichophyton mentagrophytes* (Sartori et al., 2003).

In the search for new and effective anticancer drug leads, the issues of genotoxicity and mutagenicity are of grave concern. To this end, KA was assessed for its possible genotoxic and mutagenic effects using established *in vitro* and *in vivo* models (Cavalcanti et al., 2006; Cavalcanti et al., 2010). It was found to exhibit genotoxic and mutagenic effects in human peripheral blood leukocytes, Chinese hamster lung fibroblast (V79) cells, *Saccharomyces cerevisiae* (baker’s or brewer’s yeast), and mice (Cavalcanti et al., 2006; Cavalcanti et al., 2010). These effects were presumed to be probably the result of either DNA-strand breaks or topoisomerase I inhibition or both (Cavalcanti et al., 2010). It was suggested that the double bond at the C-16 moiety might be active site responsible for the genotoxicity of KA (Cavalcanti et al., 2010). Alongside thirteen other natural isolates, KA was found to exhibit considerable antiproliferative effects in five cell lines, HeLa, A-549, Hep-2, PC-3, and MCF-7 cells in a dose-dependent manner (Cuca et al., 2011). Alves Â et al. (2023) in their bid to circumvent the hydrophobicity and thermosensitivity challenges of KA, prepared complexes of *ent*-kaurenoic acid-enriched *Mikania glomerata* leaves extract with β-cyclodextrin and assessed the antitumor activity of this formulation in rodents. The formulation displayed low systemic toxicity in mice and its antitumor activity was ascribed to its ability to inhibit LDH activity and NF-κB signaling pathway (Alves Â et al., 2023). Antitumor activities have also been reported for microbial-derived KA derivatives against the breast cancer cell lines, MCF-7 (da Costa et al., 2018) and 4 T1 (Ferreira et al., 2022), the human glioblastoma cell line, U87 (Lizarte Neto et al., 2013) and other cell lines (Dutra et al., 2014).

Other reported biological activities of KA include hepatoprotective (Marcondes-Alves et al., 2019), leishmanicidal (Miranda et al., 2015), smooth muscle relaxant (de Alencar Cunha et al., 2003), trypanocidal (Kian et al., 2018), vasorelaxant (Tirapelli et al., 2004), anticonvulsant effect (Okoye et al., 2013), and hypoglycemic (Raga et al., 2010) effects among others.

### Xylopic acid (XA)

A perusal of extant scientific literature on XA (15β-acetoxy-*ent*-kaur-16-en-19-oic acid) reveals reports on the pharmacokinetics and *in vitro* microsomal liver enzyme metabolism (Alolga, et al., 2023a), forced degradation studies (Alolga, et al., 2023b), quantitative analyses (Adosraku & Oppong Kyekyeku, 2011; Kyekyeku et al., 2020), semi-synthesis (Soh et al., 2022) and evaluations of diverse biological activities. The anti-inflammatory potential of XA has been assessed using various animal models and found to be effective against acute and chronic inflammation (Osafo et al., 2018; Osafo et al., 2019). For instance, Osafo et al. (2018) found XA to be effective against acute inflammation and this effect was due to its ability to modulate the effects of pro-inflammatory markers, prostaglandin E2, serotonin, histamine, and bradykinin. The possible mechanisms that underlie the anti-inflammatory effect of XA according to Boakye et al. (2022) could be due to its ability to regulate the activities of Nrf2 and NF-κB together with increase in HO-1 expression and reduction in VCAM-1 expression. Against chronic inflammatory conditions such as rheumatoid arthritis (RA), XA was found to ameliorate the inflammatory states of adjuvant-induced arthritic rats via reduction of pro-inflammatory cytokines (IL-6 and TNF-α) levels (Osafo et al., 2022). As the principal constituent of a bioinspired reconstituted high-density lipoprotein (rHDL) nanoparticles, the anti-RA potential of XA was assessed via the lens of metabolomics and transcriptomics (Alolga et al., 2021). The anti-RA activity of the rHDL/XA nanoparticles was due mainly to the restoration of perturbed metabolic pathways, thus, amino acids and lipids metabolism (Alolga et al., 2021).

Other bioactivities reported for XA include antimalarial (Boampong et al., 2013; Ameyaw et al., 2018; Osei et al., 2021), antimicrobial (Boakye-Yiadom et al., 1977), antinociceptive (Woode et al., 2015), analgesic (Woode et al., 2013; Ameyaw et al., 2014; Woode et al., 2016), antiproliferative (Soh et al., 2022), antidepressant-like (Biney et al., 2021), and cardiovascular and diuretic (Somova et al., 2001) effects.

### Call for further research

The ultimate objective of scientists interested in bioprospection for lead compounds has always been to discover new and more effective drugs for the numerous diseases that have plagued humanity. However, the journey from translation of laboratory findings to clinical application is a herculean task. It involves years of preclinical studies, much of the outcome of which usually fails even before clinical trials. As far as the *ent*-kaurane diterpenoids are concerned, there is dearth of research on their bioactivities. Much of the research done has been to isolate and structurally elucidate these compounds from their respective plant sources. Isolation and structural elucidation of compounds is merely the first step to a long and winding journey towards possible clinical use. With the advent of computer-simulated combinatorial chemistry and high-throughput screening techniques, there is need for more attention to be devoted to research on the bioactivities of the *ent*-kaurane diterpenoids. These techniques in combination with established *in vitro* and *in vivo* models would aid in the discovery of lead compounds for probable clinical trials. In-depth investigations of the most active compounds would elucidate their exact molecular mechanisms of actions, pharmacokinetic and toxicological profiles. Further research on the most active compounds would also identify and resolve bioavailability and formulation challenges prior to clinical trials. The possible medical solutions to inflammation-related chronic diseases such as diabetes, RA, ulcers, cancers, and even age-long diseases such as HIV/AIDs and malaria, could lie in the *ent*-kaurane diterpenoids based on the results of available preliminary investigations.

## Conclusion

This mini-review provides to a large extent a summary of research progress on the *ent*-kaurane diterpenoids isolated from various plants in the Annonaceae family, highlights the reported biological activities of these compounds and proffers suggestions for future research on same. In summary, the *ent*-kaurane diterpenoids are a group of compounds with a probably huge potential as good drug leads but have not had much attention from the scientific community. Available data on preliminary studies conducted on these compounds have credited them with diverse pharmacological properties including but not limited to antimicrobial, anti-inflammatory, anti-HIV, leishmanicidal, trypanocidal, and antimalarial effects among a host of others. There is however a need for further research so as to fully tap into the potential medical benefits of these compounds.
