# Supplementary material for: Ent-kaurane diterpenoids from the Annonaceae family: a review of research progress and call for further research
Source: Front Pharmacol. 2023 Jun 29;14:1227574. doi: 10.3389/fphar.2023.1227574 (PMC10345206; doi:10.3389/fphar.2023.1227574)
Supplement: Supplementary file 1 [file DataSheet1.docx]

**SUPPLEMENTARY MATERIAL**

**MINI-REVIEW**

***Ent*-kaurane diterpenoids from the Annonaceae family: A Review of Research Progress and Call for Further Research.**

Traore S. Ibrahim^1a^, Purevdulam Khongorzul^1a^, Moses Muyaba ^2^, Raphael N. Alolga^1*^

^1^State Key Laboratory of Natural Medicines, Department of Pharmacognosy, School of Traditional Chinese Pharmacy, China Pharmaceutical University, Nanjing 211198, China.

^2^Department of Pharmaceutical Chemistry and Pharmacognosy, School of Pharmacy, Eden University, Great East Road Campus, Lusaka, Zambia.

**^a^** Authors share first authorship

^*^ **To whom correspondence should be addressed:**

Raphae N. Alolga, PhD, State Key Laboratory of Natural Medicines, Department of Pharmacognosy, China Pharmaceutical University, Nanjing, China. Email: [alolgara@cpu.edu.cn](mailto:alolgara@cpu.edu.cn) Tel.: +86 137 7655 0643


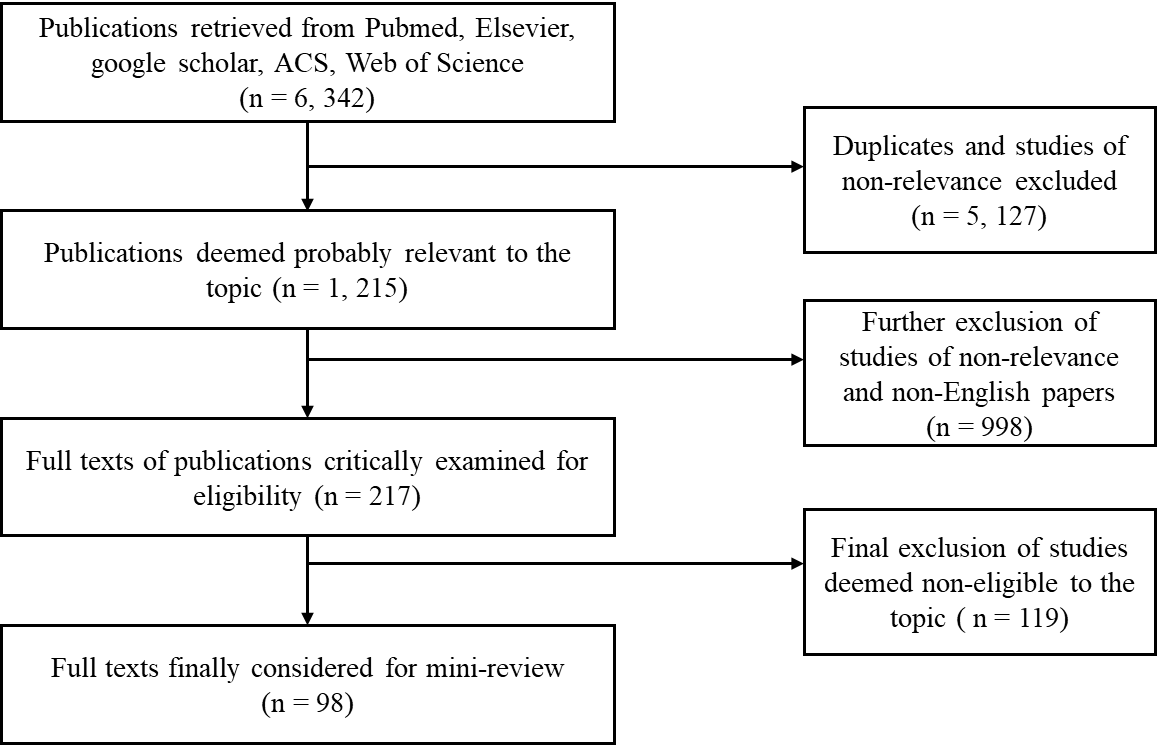


**Supplementary Figure 1.** A flow chart summary of how the studies used for the mini-review were selected.

**Supplementary Figure 2**. Chemical structures of non-dimeric *ent*-kaurane diterpenoids isolated from plants in the Annonaceae family. Identities of all compounds (1-67) are indicated in Table 1.

**Supplementary Figure 3**. Chemical structures of kaurenoic acid, KA **(A)** and xylopic acid, XA **(B).**
